# Examining the use of complementary and alternative medicine among older persons in Ebonyi State, Southeast Nigeria: a qualitative study

**DOI:** 10.1186/s12906-026-05390-7

**Published:** 2026-04-29

**Authors:** Chibuike Innocent Agu, Ugenyi Victoria Iloabachie, Onyinyechukwu Uzoamaka Oka, Irene Ifeyinwa Eze, Lenz Nwachinemere Okoro, Edmund Ndudi Ossai

**Affiliations:** 1 Health Policy Research Group, University of Nigeria Nsukka, Enugu Campus, Nsukka, Enugu State Nigeria; 2 Department of Community Medicine, Alex-Ekwueme Federal University, Ndufu-Alike, Ebonyi State Nigeria; 3 Department of Community Medicine, College of Medicine, University of Nigeria Nsukka, Enugu Campus, Nsukka, Enugu State Nigeria; 4 Department of Community Medicine, College of Medicine, David Umahi Federal University, Uburu, Ebonyi State Nigeria

**Keywords:** Complementary and alternative medicine, CAM, Older persons, Healthcare use, Qualitative study, Ebonyi State, southeast Nigeria

## Abstract

**Background:**

Despite the popularity of complementary and alternative medicine among older persons, its use within this subpopulation is still not fully understood. This study aimed to explore the perceptions of older persons residing in communities in Ebonyi State as pertains the use of complementary and alternative medicine.

**Methods:**

Qualitative data collection was by focus group discussions (FGDs), in four communities, two urban and two rural, in Ebonyi state, Nigeria. A total of 12 FGDs involving 96 participants, each consisting of 8 males or females respectively were conducted using a synthesized FGD guide. A thematic analysis of data was performed with the aid of NVivo software.

**Result:**

The Leventhal’s self-regulatory model (SRM) was utilised as it provides a flexible framework for understanding the use of complementary and alternative medicine among older persons. Majority of the participants expressed belief in the inherent benefits of complementary and alternative medicine, which were categorized into medical and non-medical reasons. Febrile illness, including malaria, typhoid fever, and hepatitis; respiratory; haematological; and dermatological conditions were reported as the common health conditions for which participants used CAM. Additionally, participants mentioned using CAM for chronic conditions, such as diabetes mellitus, hypertension, and arthritis, as well as for general health promotion and wellbeing. Preference for CAM was influenced by belief in its effectiveness, perceived lower cost when compared to conventional treatments, delays in hospital diagnoses and treatments, and belief in the spiritual origins of diseases. Safety concerns regarding CAM use included a lack of information on dosing, directions for safe use, and potential side effects.

**Conclusion:**

The findings indicate a strong belief among participants in the benefits of complementary and alternative medicine which they believe offer both medical and non-medical advantages. However, despite these perceived benefits, safety concerns were also raised by some participants. Efforts to promote education and awareness about CAM, improve access to reliable information, and ensure the safe and effective use of CAM therapies are crucial for supporting the health and well-being of older persons who choose to incorporate CAM into their healthcare practices.

**Supplementary Information:**

The online version contains supplementary material available at 10.1186/s12906-026-05390-7.

## Background

There has been an increasing trend in the use of various alternative medicine approaches in many countries in recent years. As an important part of health services, these practices, treatments and technologies popularly known as complementary and alternative medicine (CAM) have grown in popularity in the last few decades, even though, there is limited research data on their safety, efficacy and quality [1, 2]. Global interest in this approach is evident in the significant use reported in countries such as Belgium (66–75%), France (49%), Australia (46%), America (34%), the UK (33%), Germany (20–30%), and the Netherlands (18%) [3]. In developing countries, alternative medicine is the primary source of health care for up to 80% of the population, and sometimes, may be the only accessible health care resource in those settings [1]. In Nigeria and other parts of West Africa, CAM, especially herbal remedies and spiritual healing are particularly common [4–6]. An earlier study in Enugu south-east Nigeria reported that 84.7% of the participants had used CAM at some point [5].

There is no globally accepted definition for CAM, but the United States Center on Complementary and Integrative Health has defined it as a group of diverse medical and health care practices and products that are not presently considered to be part of conventional medicine [7]. In Nigeria it has been described as any treatment modality, option or service not often prescribed by orthodox medical doctors or taught in conventional medical schools [8]. They include options such as native herbs, garlic and medicinal teas; nutritional supplements; acupuncture; massage; meditation/yoga; spiritual therapy such as prayer and spiritual healing; body scarification and traditional surgery such as traditional bone setting [8]. These therapies are referred to as alternative medicines when used in place of conventional treatments, and complementary when used alongside conventional treatments [7].

Previous research show that these treatment modalities are used by all age groups for a wide range of conditions, including non-communicable diseases, psychological conditions such as anxiety and depression, and life-threatening illnesses including cancer and HIV infection [9–12]. It is interesting to note that CAM use tends to be more common among individuals who have more than one medical condition, and patients with cancers and other chronic diseases [10, 13]. As the older persons are more susceptible to chronic health conditions due to the natural ageing processes, CAM has gained popularity with many older persons and their caregivers [14]. An analysis of the European social survey data from 21 countries showed that a third of older Europeans experiencing musculoskeletal pain reported using CAM within the 12 months preceding the study, as against nearly 88% of older Americans who were reported to have been using CAM according a Health and Retirement Study [15, 16]. Similarly, evidence from different parts of Nigeria suggests that an older age group is associated with increased CAM use [5, 6, 17].

It is often believed that CAM offers a gentler and safer approach to addressing common health conditions suffered by older persons, and that CAM plays a substantial role in health promotion, as well as treatment and prevention of diseases and frailties in older persons, which encompasses the wholesome approach of handling ageing and its effects [18, 19]. Thus, they often incorporate alternative and complementary therapies into their health self-management routines, either as self-care behaviours, informal support, formal support, or medical care [20]. There is a global trend of demographic ageing, an evidence that the onset of morbidities is being postponed and compressed into a shorter period of older age, and a change in disease patterns leading to the high rates of CAM use among older persons [21]. Nigeria has the highest number of older people in the continent and the 19th highest globally, with the population of Nigerians aged 65 and older projected to triple by 2050, the use of CAM by older persons in the country is still not receiving appropriate attention [22]. Considering this, and the limited empirical evidence of efficacy for many CAM treatments, it is important to gain a better understanding of CAM is perception and utilization by older persons. The findings from this study will provide the evidence base for health planners and policy makers in designing policies and programmes aimed at improving the health of older persons.

### Theoretical framework

Leventhal’s self-regulatory model (SRM) provides a theoretical framework for understanding the use of complementary and alternative medicine among the older persons [23]. According to this framework, individuals’ engagement in specific self-management behaviours, such as CAM use, can be attributed to several factors. These include beliefs about a symptom, state, or illness (individuals’ beliefs about their symptoms or health conditions) play a significant role in determining their self-management behaviours. Older persons may turn to CAM therapies based on their perception of the effectiveness of these treatments in addressing their health concerns) [24]; their perceptions and understanding of their health (older persons who perceive themselves as having poor health or who believe in the holistic principles of CAM) may be more inclined to explore alternative treatment options); their knowledge of treatments (to use CAM, individuals need to know them and their potential benefits) [25]; personal resources (individuals resort to CAM therapies which are perceived as affordable); and structural factors that affect access to a therapy (older persons are more likely to use CAM if they face barriers to accessing conventional healthcare services or if CAM is readily available and culturally accepted within their communities) [26].

## Methods

### Study design and study population

This was a cross-sectional qualitative study. A total of 12 focus group discussions (FGDs), each consisting of either 8 males or females were conducted using a synthesized FGD guide (see supplementary file). Thus, the study consisted of 96 participants, comprising 48 male and 48 female older persons (aged 65 years and above) who resided in the selected urban and rural communities of Ebonyi State, Nigeria.

### Sample selection

A multi-stage sampling method was used in the study. In the first stage, the thirteen LGAs in Ebonyi State were stratified into urban and rural local government areas, and a simple random sampling technique was used to select two local government areas each from the urban and rural areas of the state. In the second stage, two communities were selected from each of the selected local government areas using simple random sampling technique. Participants were then purposively selected based on their age, sex, and highest level of education, and the FGDs were conducted with the different groups until data saturation was achieved.

### Data collection

Detailed information on the types of CAM used, health problems for which they are used, other reasons for CAM use, and cost of CAM were collected. Before data collection, the FGD guide was pretested in two different communities not selected for the study. Deficiencies or ambiguities of the study instrument which were detected were amended. The discussions were held in secluded places like public primary schools and community town halls, and in English or Igbo language, depending on participants’ preferences. Each session was facilitated by a moderator, a note-taker, and a local guide/translator, and lasted between 45 and 60 min each. All FGDs were recorded manually and using audio recorders with the permission of the participants.

### Data analysis

The recorded discussions of focus group discussions were transcribed verbatim following each session by note-takers. All the discussions in Igbo language were translated into English. For quality assurance purposes, the scripts were compared with the written notes for completeness and accuracy. The notes were used to assign proper labels to the transcripts, and to further enrich the transcripts with nuances and non-verbal cues observed during the sessions. Coding of transcripts was based on themes as they emerged during the coding process. The themes from each discussion were reviewed by the researcher and grouped under wider themes. Thematic analysis of transcripts was performed using deductive-inductive approach, and with the aid of NVivo software. The results are structured according to four themes, which include perceived health benefits of CAM use, preference for CAM, belief in the spiritual origins of diseases, and safety concerns about CAM.

### Study area

The study area was Ebonyi State, one of the five states in southeast geo-political zone of Nigeria. It is located on latitude: 6° 15’ 18” N, longitude: 8° 05’ 55” E. It is bordered by Benue State to the north, Enugu State to the west, Imo and Abia States to the south and Cross River State to the east [27]. It is made up of thirteen local government areas, three of which are urban while ten are rural. The state is inhabited mainly by people of Igbo ethnic nationality and their main language is Igbo. The literacy level of the people is 70% [28]. It has a land area of 5,533 square kilometers and a 2022 projected population of 4, 143, 100 people, based on 2006 population census, with older persons constituting 3.1% of the population [29]. Being an agrarian entity, the occupation of Ebonyi people is predominantly farming supported with over 3525km^2^ of very arable land, 75% of which is upland and 25%, swamp. This enhances growing of variety of cash and food crops, such as, rice, yam, cassava and also animal husbandry. Other occupation of Ebonyi people are civil service, trading and unskilled labour. Health services are provided through public and private health facilities in the State. Additionally, there are pharmacy premises, patent medicine, and grocery stores, which can be common sources of CAM for the people [30, 31].

## Results

### Interviewers’ characteristics

The FGDs were conducted by the researchers who are fellows of the West African College of Physicians, ENO is a Senior Lecturer at Ebonyi State University Abakaliki, Nigeria while CA is a Consultant Public Health Physician at Alex-Ekwueme Federal University Hospital, Abakaliki, Nigeria. Both are males and have requisite skill and experience in conducting qualitative research.

### Participants’ profile

The age range of participants for focus group discussion in the urban area was 65 to 88 years while, in the rural area, it was 65- 85years. A total of 48 males and 48 females participated in 12 FGDs. Half of the urban participants had either completed secondary (24.7%), or tertiary education (25.3%), while most of the rural participants (66.7%) had no formal education. Most were either retired or unemployed (48.0%), about one-third (32.3%) were self-employed, while the rest were on salaried employment (19.7%).

#### Perceived health benefits of CAM use

Majority of the study participants believed that there were several health benefits inherent in the use of CAM. They believed that CAM could be effective in treating a wide range of health conditions. They included acute illnesses, such as febrile conditions (e.g., malaria, typhoid), and dermatological (eczema, acne) and haematogical disorders; non communicable diseases (hypertension, diabetes mellitus).

##### Use for acute illnesses

**Febrile conditions**

Among the participants, the commonest condition for which CAM was used was febrile illness, ranging from malaria, typhoid to viral hepatitis. Participants were specific about the herbs that are used for the treatment of febrile illnesses, which included mango leaves, Neem *(Azadirachta indica)*, ‘mmimi ohia’, (i.e. finger root) *(Uvaria chamae)*, Lemon juice. Some of the participants shared their experiences:

*“Yes mango tree is also used by us for malaria treatment; just scrap the back*,* collect lime*,* slice it*,* peel and put lemon grass. If you drink this for malaria you will certainly recover from it.”* (Male participant, urban).

*“If you have typhoid fever*,* or ‘iba ocha n’aya’ (meaning hepatitis)*,* just collect guava leaves*,* Mgbegbe and dogonyaro (Azadirachta indica)*,* wash off dirt from them and cook them. Bring it down when it is still very hot*,* cover yourself with wrapper. The heat will penetrate into your body. When it gets cold*,* the person will still bathe with the water. It works very well for the two conditions.”* (Female participant, rural).

**Respiratory conditions**

Another group of medical conditions for which participants used CAM for treatment were respiratory conditions. These conditions included those resulting in cough, catarrh, nasal discharge/blockage, hoarseness of voice, and even breathlessness. Which may also include, rhinitis, asthma, bronchitis, laryngitis and pneumonia. Participants’ experiences regarding the treatment of respiratory conditions are expressed:

*“Extracts from scent leaves are commonly used in the treatment of catarrh*,* nasal congestion and other forms of respiratory challenges in this community. For instance*,* it can be rubbed between the palms*,* sniffed and there is instant relief from nasal blockage”.* (Female participant, rural).

*As I was talking about that* ‘utazi’, *(Gongronema latifolium)*,* and* scent *leaf (Ocimum gratissium)*,* we usually squeeze them together and drink the extract against cough. My husband used it towards the end of last year*,* around October and November; that time*,* cough (Covid − 19) disturbed people so much.* (Female participant, urban).

**Dermatological conditions**

Another participant gave further insights concerning the use of CAM in the management of certain dermatological health conditions. Some of these include allergic dermatitis, chicken pox, eczema, and dandruff.

*“Concerning skin diseases*,* I have used crushed onions on some rashes and they were gone. Also*,* aloe Vera works wonders if used on dandruff. These are the two I have actually tried and they worked for me.”* (Female participant, urban).

**Haematological conditions**

Additionally, few participants had used CAM for other medical reasons, such as in the treatment of haematological conditions (anaemia, blood loss etc.), as well as for health promotion and wellbeing.

*“There is an herb called physic plant (Jatropha curcas). It is used to stop bleeding in our community for people that have problems with blood clotting. Whenever someone has a cut and it is squeezed and the extract dropped on the cut the bleeding stops immediately. i.e for people whose blood does not clot well*,* so*,* it is used to arrest bleeding”.* (Male participant, urban).

*‘Egbaroko’ khaya ivorensis is a blood supplier. If you collect it and bring out the extract by crushing it*,* mix it with malt*,* it gives blood very well*,* so it is used to treating people with symptoms of anenaemia.* (Male participant, rural).

##### Use for chronic diseases

A lot of the participants also utilized CAM products for the management of non-communicable diseases, such as arthritis, hypertension, and diabetes. The majority, particularly, in the rural areas used it in the treatment of arthritis. Some of the participants gave specific details regarding the CAM they used in treating these medical conditions:

**Arthritis**

The commonest non-communicable disease being treated using CAM especially among the participants was arthritis. This was more emphasized in the rural communities.

*“Arthritis was a major problem to me*,* but after my son took me to where I did ‘acupuncture’ and thereafter*,* started using forever living product*,* I have not been having that challenge again. I can even climb my staircase these days unlike when I was using ‘oyibo’ medicine (orthodox medication) from the chemist (patent medicine vendor).”* (Female participant, rural).

**Diabetes mellitus**

Secondly, was diabetes mellitus. However, this was more common among the urban dwelling participants.

*“I have been a diabetic for a long time and what has been very good at controlling my sugar level has been an herbal product which I prepare by myself. It is made up of scent leaf*,* bitter leaf*,* pumpkin leafs squeezed together and the extract filtered into a pot for boiling. After cooling*,* I take it morning and night.”* (Male participant, urban).

**Hypertension**

A few of the participants also utilised CAM for the management of hypertension

*“I was on an antihypertensive and it was really giving me concern as I always had severe headache while taking the medicine. However*,* a friend introduced me to the use of olive oil*,* which I use now as it controls my blood pressure.* (Female participant, urban).

##### Use for health promotion and wellbeing

Furthermore, many participants highlighted the role of CAM in promoting overall health and wellbeing, beyond just treating specific diseases. The participants explained that herbs could be used to maintain good health. A participant made his views known in the following statement:

*“There is this herb or vegetable we call ‘Ugu’*,* Telfairia occidentalis. If it is collected*,* and boiled with water*,* add tomatoes and mix. It helps to keep someone healthy.” Anyone who takes it regularly will hardly become sick.* (Male participant, rural).

#### Preference for CAM

##### Belief in CAM effectiveness

The commonest non-medical reasons, particularly among the urban participants, for the preference of CAM in disease treatment is the belief in its effectiveness.

*“I wake every morning*,* I collect uvuru (Nauclea latifolia). There was a time I had headache and fever and I realized that all the drugs from the chemist was not helping me*,* so I resorted to uvuru and it took care of my health. I do it every early morning. I buy fifty naira uvuru and drink.* (Male participant, urban).

*“Ginger and honey are also important*,* any cough that has resisted drugs cannot resist ginger and honey mixed and taken together. They clear all types of cough very well”.* (Female participant, rural).

*“There is a plant called ‘Ahuji’*,* (scent leaf) that can cure what conventional/orthodox medicine or drugs can’t cure. If the leaf is chewed*,* it will bring out the real symptoms of what is happening to you and then cure the disease in a wonderful way”.* (Male participant, urban).

The view that CAM products were more effective than orthodox medicine was supported by a participant in the rural study group, who even asserted that he had stopped taking orthodox medicine because of his past experience with it. He expressed his sentiments in the following manner:

*“I don’t take orthodox medicine. I depend so much on alternative drugs because that is what has kept me alive today*,* with God helping me. I had an ailment that threatened my life*,* and I had up to four investigations in a hospital*,* while my health deteriorated. It was this alternative medicine that finally cured me of the ailment.”* (Male participant, rural).

However, one of the participants in the urban community had a different opinion on the effectiveness of CAM. He argued that orthodox medicine was more effective than CAM, but that his ability to seek treatment was based on his financial capability which affects his health seeking behaviour. He made known his thoughts this way:

*“I know that orthodox medicine work very well even in comparison with some alternative medicine*,* but the main reason for going for alternative medicine is due to lack of money. If you are sick and you have money obviously you will go to a hospital. Whatever they (health providers) tell you to do*,* you do*,* and you will be okay; if it is orthodox medicine*,* it works sharp*,* sharp”* (Male participant, urban).

##### Perceived lower cost of CAM

Conversely, most of the rural participants cited lower cost of CAM as the major reason for opting for CAM treatment. Typical responses from the participants were:

*“There is an extent to which an illness could disturb one*,* and you don’t have money*,* you go to the village and collect leaves around you and cook. So*,* there are times one resorts to alternative medicine due to lack of money”* (Female, participant rural).

*“My last comment or word concerning this thing we have discussed now*,* is that our people say that*,* ‘a normal person does not expose his eyes to fire’. Money is just the challenge. If there is money*,* even common headache will make one go to the hospital*,* but if you don’t have money you come home and sort yourself out with alternative medicine which costs little or nothing*,*”* (Male, participant rural).

#### Delay in hospital diagnoses/treatments

Other important reasons given for the preference of CAM use to orthodox medicine in treatment of diseases were “delay in hospital diagnoses/treatments” and the belief that “CAM products act more rapidly than orthodox medicines.” These viewpoints were expressed by urban and rural respondents respectively. Their perspectives were presented in the following quotes:

*“If you go for orthodox medicine*,* especially in the hospital*,* they will ask for test (laboratory investigations)*,* the result of which may take time to come out*,* sometimes*,* even days*,* but if it is alternative medicine*,* no time is wasted; just bring certain leaves (herbs)*,* prepare them and take*,* and you will be cured.”* (Female participant, urban).

*“A sister*,* in fact that man’s wife*,* (pointing at a participant) was sick and nearly died. We went to the teaching*,* hospital*,* they did not do much*,* but kept giving one appointment after the other*,* eventually*,* we got alternative medicine which the woman took*,* and she is now healthy”.* (Male participant, urban).

##### Belief that “CAM products act more rapidly than orthodox medicines

*“Let me say something on another reason for using it (CAM). It is not just because it is cheap or because we do not have money*,* but also*,* because it (CAM) is faster in action when compared to the other (orthodox) medicine. Just like what this woman said*,* somebody can go to hospital many times for an issue*,* without any improvement. So*,* for me*,* it is about the very rapid action of CAM that I use them”.* (Female participant, rural).

*“Sometimes people would go to hospital severally for a particular issue without any improvement*,* then they will resort to alternative medicine and get well. That is what makes people to go for CAM”* (Female participant, rural).

#### Belief in the spiritual origins of diseases

##### Belief in rituals as solution to some spiritual conditions

Moreover, few participants believed that certain diseases had spiritual origins and were better treated with some forms of CAM, such as rituals. Here are the expressions of these participants:

*“Last week*,* I was sick*,* and I was prepared to go to hospital but was hindered by some obstacles. I coughed and there was blood in my sputum. I met a herbalist that gave me ‘Nka’ to chew. I chewed it and got healed.”* (Male, participant, urban).

*“There are some symptoms you will have*,* and it will not be wise to rush off to a hospital. In such a situation*,* it will be better to first of all*,* take some local medicine and rule out some spiritual diseases.”* (Female, participant, urban).

##### Belief in prayer as sole remedy for some spiritual conditions

However, two participants hinted that certain health conditions were only amenable to prayers. Their views were captured thus:

*“Sometimes*,* both CAM products and conventional medicine will fail*,* and only prayer will be used to cure the disease concerned. You can also*,* say only prayer and you get cured/relieved.”* (Female participant, urban).

*“What I am saying is that sometimes poison can resist all manner of drugs*,* (both orthodox and CAM)*,* but if you go for prayers*,* you receive your healing.”* (Male participant, rural).

### Perceived dangers of conventional therapy for a spiritual condition

Some participants expressed the view that administering orthodox medicine, particularly injections to patients with such diseases would result in their death.

*“That thing called ‘oke ezenwu’*,* (a disease condition) is not cured in the hospital. Some people with the condition*,* may go to a hospital without knowing that they have the disease. If they take orthodox medicine*,* they will die from the disease. It is only ‘Igbo’ medicine (referring to herbs) that cures the sickness*,* perhaps with ritual performance.”* (Male, participant, rural).

*“It is not every disease that is cured with modern medicine. For example*,* there is a particular boil (furunculosis) that can be on the body or inside the body*,* if somebody with it mistakenly goes to a hospital or chemist and is given injection*,* the person will simply die.”* (Male, participant, urban).

### Safety concerns about CAM

#### Lack of information on dosing and directions for safe use

Despite the widespread use of CAM among both the urban and rural participants, and the various views expressed in support of the preference for its use, most of the participants in the study agreed that there were crucial issues surrounding the safety of some of the CAM products, particularly, the unprocessed herbal preparations. They opined that most CAM products had no information on dosage, potential side-effects, and directions for safe use. A rural participant made his point in this quote:

*“The challenge is that most CAM products don’t have dosage*,* especially the local herbs or direction on usage. One just takes it anyhow*,* but if it were the orthodox medicine*,* you know what quantity at any point in time*,* and even if you don’t know how to read*,* someone can help you with the written prescription.”* (Male participant, rural).

Two urban participants declared that they did not take alternative medicine because of CAM products had no dosages. One of the participants even went further to advocate that herbs should not be taken. Their views are captured thus:

*“What I am saying is that anything that doesn’t have dosage should not be taken because those alternative medicines are just*,* O’ God help me*,* (Laughs). You don’t have information on what you take or how much of it to take.”* (Male participant, urban).

*“This issue of alternative medicine; there are people who take it but we can’t say if it is good or not*,* but I don’t take it because it doesn’t have dosage. That is why I don’t take it.”* (Male participant, urban).

#### Potential side-effects

Other adverse effects attributed to CAM by the participants were liver or other gastro-intestinal problems, renal pathology and even death associated with the use of some form of CAM.

*“There are some alternative medicines*,* such as the one called ‘Nchala’ that causes passage of watery stool on consumption*,* and if not controlled with something like palm oil it may lead to the death of the person.”* (Female participant, rural).

*“Alternative medicine can cause liver problem because it does not have dosage guide. No instruction on how to use it; some have taken it and had their abdomen swollen due to liver or kidney damage”* (Male participant, urban).

## Discussion

This study explored the use of complementary and alternative medicine among the older persons in communities in Ebonyi State, Southeast Nigeria. It showed that almost all the participants believed that there are health benefits associated with the use of CAM. This is similar to findings from a study in Australia [25]. This widespread belief underscores the importance of CAM in the healthcare landscape and suggests a high level of acceptance and trust in its potential efficacy among the participants. In contrast, a study in Enugu, south east Nigeria observed that only a quarter of the respondents could describe specific benefits from the use of CAM [32]. This discrepancy might be due to differences in the methodologies of the studies.

The finding that the commonest condition for which CAM is used is febrile illness, ranging from malaria, and typhoid fever to hepatitis is contrary to the results from a study in Saudi Arabia, which reported musculoskeletal disorders, cardiac problems, and neurological problems as the primary health reasons for CAM use among older persons [33]. The difference in findings may be due to variations in disease patterns between the regions, and this may be attributed to various factors, including environmental conditions, and access to healthcare services. In regions, such as the study area where infectious diseases are endemic or where access to conventional healthcare is limited, individuals may turn to CAM for managing acute illnesses such as febrile conditions. On the other hand, in more developed climes with aging populations, chronic conditions like musculoskeletal disorders and cardiovascular diseases may be more prevalent, driving the utilization of CAM for symptom management and holistic care.

The findings from this study suggest that older persons commonly turn to CAM due to two primary non-medical reasons which are belief in its effectiveness, and lower costs. These findings further affirm an earlier study in another part of the state which reported that most older persons preferred alternative medicine to conventional medicine due to presumed efficacy and cost among other factors [24]. Also, the findings are consistent with similar studies conducted in Bangladesh, and Germany [34, 35]. The belief in the effectiveness of CAM reflects a common perception among the older persons that these modalities offer therapeutic benefits beyond those provided by conventional medicine. This belief may stem from personal experiences, or cultural traditions, as many older adults have lifelong experience with alternative medicine due to unavailable conventional care during their childhood [36]. In addition, the perception of lower costs associated with CAM aligns with global trends of seeking more affordable healthcare options, particularly among older adults who may be facing financial constraints.

Another rationale for the use of CAM by older persons as observed from this study is dissatisfaction with conventional health service delivery. There was a perception among most of the participants that there is poor quality of care in orthodox healthcare settings, such as delay in diagnoses and treatments. Such people believe that traditional healthcare services are quicker, and that CAM products act more swiftly compared to orthodox medicines. This further affirms the findings from previous studies in sub-Saharan Africa that one of the major reasons for non-use of health facilities is poor quality of care coupled with attendant delays in accessing care, often attributed to drug and equipment shortages, under-staffing, or poor infrastructure and poor attitudes among health workers [37–39].

Further, few of the participants believed that certain diseases have spiritual origins and were better treated with some forms of CAM, such as rituals. Earlier studies in different parts of the country reported that certain disease conditions are considered incurable with western medicine because of belief in their spiritual origins and, as such, are only treated with alternative medicine [40, 41]. This finding is not unique to Nigeria, but was also reported in various parts of both the developed and the developing nations [42]. This finding reflects a cultural and religious perspective on health and illness that is common in many societies. The notion that certain diseases can only be addressed through alternative therapies due to their spiritual origins underscore the complexity of health beliefs and practices in the region. In Nigeria, several people believe that prayer alone can effectively address all their challenges, including sickness. As a result, a large number of people attend miracle healing services seeking relief from various health conditions [43]. This highlights the significant influence of religious beliefs on health-seeking behaviours, and the need to understand them in healthcare provision.

In spite of the widespread use of CAM among the participants, few participants raised concerns about lack of appropriate dosing and potential side effects. The finding is similar to a previous study carried out in Lagos, southwest Nigeria, and this suggests that the issue may not be an isolated one, but rather reflects a broader pattern across different regions of the country [17]. This lack of dosing could potentially lead to misuse or overuse of alternative therapies, which in turn may increase the risk of adverse effects. Thus, efforts should focus on promoting evidence-based practices, and strengthening regulatory measures to ensure the safe and effective use of alternative therapies.

The major limitation of this study is that generalizability is limited, since this is a qualitative study. However, the use of FGD enabled an in-depth exploration of the perspectives of older persons residing in these communities, while data synthesis was based on Leventhal’s self-regulatory model which provided a theoretical framework for understanding the use of CAM among the older persons. Future research should explore the efficacy and safety of CAM therapies and their integration into mainstream healthcare practice.

## Conclusion

The study findings indicate a strong belief among participants in the benefits of complementary and alternative medicine which they believe offer both medical and non-medical advantages. The use of CAM was prevalent among the participants particularly for managing febrile illnesses like malaria, typhoid, and hepatitis, as well as for other health conditions, such as respiratory, haematological and chronic conditions. Their preference for CAM were influenced by factors, such as perceived effectiveness, cost advantages compared to conventional medicine, delays in hospital treatments and belief in the spiritual origins of diseases. Despite the perceived benefits of CAM, safety concerns were also raised by some of the participants. These include lack of information on dosing, directions for safe use, and potential side effects associated with CAM therapies. The findings underscore the interplay of beliefs, preferences, and safety considerations surrounding the use of CAM among the participants.

Based on the findings of this study, the following recommendations are made.

To the government:

- In order to guarantee safety, effectiveness, and responsible usage, the state must develop appropriate policies on goods used in complementary and alternative medicine.
- Also, for the regulation of the sale of CAM products in the state to assure the authenticity of advertised items.
- The State government should implement the provisions of the 2019 medicine and associated product advertisement laws to make sure that the public is only provided with accurate, comprehensive, and verified information about CAM.
- The State government should develop policies for the care and well-being of the older persons that will guarantee the creation of a social safety net for them. Examples of these policies include drug and fee exemptions or differential charges that are directed towards the older persons who are most in need, especially those living in rural areas. (To improve older peoples’ financial security)
- To reduce older persons financial difficulty when obtaining care, community health funding should be investigated as a substitute for traditional health care financing.

With regards to the medical professionals:

- It is imperative to accord priority to the healthcare requirements of the elderly population by providing them with preferred treatment within medical facilities.
- To properly address the medical requirements of older persons, geriatric medicine subspecialties should be formed in teaching hospitals.
- To facilitate counselling, primary healthcare providers should be trained on how to elicit information complementary and alternative medicine use among their elderly patients/clients.
- Health education in the communities regarding the risks of using herbal remedies and drugs at the same time is important, given that a good number of respondents admitted to combining CAM with conventional care.

## Supplementary Information

Supplementary Material 1.

## Data Availability

Some of the data generated or analysed during this study are included in this published article. Additional data are available from the corresponding author on reasonable request.

## Abbreviations

CAM: Complementary and alternative medicine
FGDs: Focus group discussions
SRM: self-regulatory model
QDA: Qualitative data analysis
