# Supplementary material for: Examining the use of complementary and alternative medicine among older persons in Ebonyi State, Southeast Nigeria: a qualitative study
Source: BMC Complement Med Ther. 2026 Apr 29;26:209. doi: 10.1186/s12906-026-05390-7 (PMC13270619; doi:10.1186/s12906-026-05390-7)
Supplement: Supplementary file 1 — Supplementary Material 1. [file 12906_2026_5390_MOESM1_ESM.docx]

**Complementary and Alternative Medicine Use among Older Persons in Ebonyi State, Nigeria**

**Focus Group Discussion Guide**

**Introduction**

Welcome the participants and introduce the purpose of the interview. Set ground rules, and ensure of confidentiality and anonymity. Obtain consent to participate and to record the discussion.

1. Have you ever used any medicine or remedy other than that given to you by medical doctor, when you experience any health problems or health needs? (*can you share your experience?)*
2. What are the different types of alternative medicines that you know about?

*(Prompt: python fat, urine therapy, massage, etc). Probe for:*

- Which ones are used in your community? How are they used? (rubbed, swallowed, recited, etc)
- Have you used any in the last one year? (Are you using any now? If yes, which one? If not, why?)

1. What are the sources of information about alternative medicines?

(*Prompt: friends, family, radio, internet, etc*)

1. What are the different health problems for which people use alternative medicines? (what about you; for what health problems have you used CAM?)
2. What are the other reasons for which people take alternative medicine?

- Probe for perceived benefits/side effects, cost, availability, etc; how do you compare the health benefits of CAM to conventional medicine?)

1. What influenced you into using alternative medicine? (probe for culture, belief in the supernatural origin of some diseases, etc.,).
2. What do you think about cost of alternative medicines? (Are CAM products/remedies cheap; expensive; prohibitive in cost). Explain

- Probe cost for each alternative medicine mentioned.
- Compared to orthodox medicine what is your opinion concerning the cost of CAM. In your opinion, which one is cheaper?
- Is cost a major reason for using CAM. (*Please, explain*)

1. In your opinion, what adverse effects do people experience from using alternative medicines?
2. Will you recommend it to a friend or family member? Why/why not?
